# Acknowledgement to Reviewers of *Antioxidants* in 2014

**DOI:** 10.3390/antiox4010022

**Published:** 2015-01-07

**Authors:** Antioxidants Editorial Office

**Affiliations:** MDPI AG, Klybeckstrasse 64, CH-4057 Basel, Switzerland

The editors of *Antioxidants* would like to express their sincere gratitude to the following reviewers for assessing manuscripts in 2014:

Abourashed, Ehab
Adjé, Félix
Aggelis, George
Ahmed, Faiyaz
Aksakal, Ercüment
Alvarez-Suarez, José Miguel
Amarowicz, Ryszard
Andrés, A.i.
Annibalini, Giosuè
Ashida, Hitoshi
Azimzadeh, Omid
Bahorun, Theeshan
Barreiro, Sonia Losada
Barreto, Maria Do Carmo
Battino, Maurizio
Beaulieu, Michaël
Belin, Christophe
Beretta, Giangiacomo
Bernini, Roberta
Bhat, Rajeev
Birch, Edward
Böhm, Volker
Borras, Consuelo
Bradshaw, Patrick C.
Bravo-Díaz, Carlos
Bullon, Pedro
Cabrera, Carmen
Camacho, M. en C. Antonio Nieto
Cao, Wei
Carrì, Maria Teresa
Caruso, Francesco
Castro-Puyana, María
Cateni, Francesca
Cavar, Sanja
Céspedes, Carlos L.
Chemat, Farid
Cheng, Jen-Hua
Choy, Ying
Chyau, Charng-Cherng
Coelho, José A
Combet, Emilie
Cortopassi, Gino A.
Costabile, Adele
Cozzolino, Mauro
Cuman, Roberto Kenji Nakamura
Custódio, Luísa
Day, Billy
De La Cruz, José Pedro
Donsì, Francesco
Dunn, Robert O.
Durazzo, Alessandra
Esposto, Sonia
Estévez, Mario
Fassett, Robert
Fleenor, Bradley S.
Franck, Thierry J.
Franco, Rodrigo
Frank, Jan
Fraser, Paul
Fregapane, Giuseppe
Gan, Chee-Yuen
Gatz, Christiane
Gibson, Claire L.
Gibson, Deanna
Gordon, Michael
Gottschalk, Leda Maria Fortes
Grilli, Alfredo
Gupta, Vivek
Hamdy Roby, Mohamed Hussein
Hasanuzzaman, Mirza
Hasegawa, Noboru
Hauer-Jensen, Martin
Hemalatha, Siva
Hernandez, Tamara Yulieta Forbes
Holuigue, Loreto
Huang, Yu
Hüttemann, Maik
Iwasaki, Masanori
Jayasinghe, Chamila
Jirillo, Emilio
Jongberg, Sisse
Jordán, María José
Juranić, Zorica D
K Pandey, Abhay
Khowala, Suman
Kita, Agnieszka
Kocsy, Gabor
Koeppen, Arnulf
Komarov, Andrei M
Kozlowski, Henryk
Kremer, Dario
Kumar, Anil
Lachman, Jaromir
Lage, Miguel Ángel Prieto
Lalas, Stavros
Landi, Marco
Lash, Larry
Legarreta, I. Guerrero
Leite, Edda Lisboa
Li, Jian Jian
López-Jornet, Pía
Lorenzi, V
Lores Aguin, Marta
Makris, Dimitris P.
Mao, Qingcheng
Martin, Pamela M.
Mascitelli, L.
Mateos, Raquel
Matés, José M.
Matsuguchi, Tetsuya
Maurizi, Charles P.
Mazzoni, I.
Méndez-álvarez, Estefanía
Merah, Othmane
Miller, Alyson
Miller, David S.
Mirunalini, Sankaran
Mohamed Jalil, Abbe Maleyki
Mohamed, RAMDANI
Morales, Juan C.
Naito, Yuji
Nerín, Cristina
Neufeld, Howard S.
Niewold, Theo
Nino, Russo
Niwa, Toshio
Obana, Akira
Oda, Tatsuya
Odriozola-Serrano, Isabel
Olcese, James
Opara, Elizabeth I.
Osakabe, Naomi
Panfoli, Isabella
Panzella, Lucia
Paraskeva, Christakis A.
Park, Miyoung
Peiró, Sara
Pereira, Eliana
Pereira, Vanda
Pizza, Cosimo
Poerschmann, Juergen
Polikarpov, Igor
Potenza, Lucia
Prasad, Majeti Narasimha Vara
Priefer, Ronny
Proestos, Charalampos
Rai Vittal, Ravishankar
Rai, Dilip K.
Ramadan Hassanien, Mohamed Fawzy
Ramos, Sonia
Ravi, Sajja
Reddy, P. Hemachandra
Remelli, Maurizio
Rodrigues, Francisca
Rodrigues, Gerard A.
Romero, Francisco Javier
Roseiro, Luísa B.
ROUANET, Jean-Max
Routray, Winny
Sahin, Selin
Salvador, Mirian
Sarker, Satya
Schauss, Alexander G.
Schempp, Harald
Serralheiro, Maria Luísa M.
Servili, Maurizio
Shang, Fu
Shen, Chwan-Li
shen, xiangchun
Shin, Sung Chul
Shukla, Shruti
Siger, Aleksander
Simal-Gándara, Jesús
Singh, Suneel Kumar
Sola, Rosa
Sørensen, Ann-Dorit Moltke
Sousa, Carla
Spitz, Douglas
Srivatsan, Malathi
Srzednicki, George
Stagkos, Dimitri
Stamatopoulos, Konstantinos
Summo, Carmine
Szymula, Marta
Taamalli, Ameni
Thiyam, Usha
Tundis, Rosa
Ufer, Christoph
Van Duynhoven, John P. M.
VanHorssen, Jack
Varzakas, Theodoros
Verardo, Vito
Villeneuve, Pierre
Wang, Dayong
Weber, John T
Wilhelm, Ethel
Wu, Chi-Rei
Wu, Li-Chen
Yamaguchi, Fumio
Zervoudakis, George
Zhang, Longze

